# Matrine Protects Against MCD-Induced Development of NASH via Upregulating HSP72 and Downregulating mTOR in a Manner Distinctive From Metformin

**DOI:** 10.3389/fphar.2019.00405

**Published:** 2019-04-24

**Authors:** Ali Mahzari, Songpei Li, Xiu Zhou, Dongli Li, Sherouk Fouda, Majid Alhomrani, Wala Alzahrani, Stephen R. Robinson, Ji-Ming Ye

**Affiliations:** ^1^Lipid Biology and Metabolic Disease Laboratory, School of Health and Biomedical Sciences, RMIT University, Melbourne, VIC, Australia; ^2^School of Biotechnology and Health Sciences, Wuyi University, Jiangmen, China

**Keywords:** matrine, NASH, inflammation, fibrosis, HSP72, mTOR, methionine choline-deficient diet

## Abstract

The present study investigated the effects of matrine on non-alcoholic steatohepatitis (NASH) in mice induced by a methionine choline-deficient (MCD) diet and the mechanism involved. The study was performed in C57B/6J mice fed a MCD diet for 6 weeks to induce NASH with or without the treatment of matrine (100 mg/kg/day in diet). Metformin was used (250 mg/kg/day in diet) as a comparator for mechanistic investigation. Administration of matrine significantly reduced MCD-induced elevations in plasma ALT and AST but without changing body or liver fat content. Along with alleviating liver injury, matrine suppressed MCD-induced hepatic inflammation (indicated by TNFα, CD68, MCP-1, and NLRP3) and fibrosis (indicated by collagen 1, TGFβ, Smad3, and sirius-red staining). In comparison, metformin treatment did not show any clear sign of effects on these parameters indicative of NASH. Further examination of the liver showed that matrine treatment rescued the suppressed HSP72 (a chaperon protein against cytotoxicity) and blocked the induction of mTOR (a key protein in a stress pathway). In keeping with the lack of the improvement of the NASH features, metformin did not show any significant effect against MCD-induced changes in HSP72 and mTOR. Matrine protects against MCD-induced development of NASH which is refractory to metformin treatment. Its anti-NASH effects involve enhancing HSP72 and downregulating mTOR but do not rely on amelioration of hepatosteatosis.

## Introduction

Non-alcoholic fatty liver disease (NAFLD) is the most common chronic liver disease with 25% prevalence in the adults worldwide. This metabolic liver disease ranges from asymptomatic simple fatty liver (or hepatosteatosis due to excessive accumulation of triglycerides in liver) to the irreversible end stages of liver cirrhosis or functional failure (Rinella, 2015; Friedman et al., 2018). Non-alcoholic steatohepatitis (NASH) is a critical stage in the progression from the early and reversible stage of simple hepatosteatosis toward serious liver damage and fibrosis, which become irreversible by any treatment. Additionally, constant repair of the injured hepatocytes during the progression of NASH can trigger the development of hepatocellular carcinoma, which is also another end stage of NAFLD (Diehl and Day, 2017). Therefore, it is important to effectively treat NASH to prevent or delay the deterioration of NAFLD to the incurable fetal stages.

Non-alcoholic steatohepatitis is characterized by hepatic steatosis, injury (elevated liver enzymes in the circulation), inflammation, and variable degrees of fibrosis (Diehl and Day, 2017). Indeed, to effectively treat NASH, a drug must possess the therapeutic efficacy on these key endpoint pathologies: namely metabolic stress (indicated by amelioration of hepatosteatosis), inflammation and fibrosis in the liver (Diehl and Day, 2017). Currently, most investigational drugs for the treatment of NASH start from a designated cellular target, such PPARα/δ, PPARα/γ, FXR, CCR2/5, SCD-1, ASK-1, ACC, caspase inhibitor, THR-β or a combination of some of them (Friedman et al., 2018). Despite the enormous efforts, there is still no approved drug specifically for the treatment of NASH (Diehl and Day, 2017).

It is widely recognized now that NASH may result from multiple factors (or “hits”) apart from simple hepatosteatosis (first “hit”) (Tilg and Moschen, 2010). Further, multiple hits are heterogeneous among different patients and can present differently in the pathogenesis of NASH (Suzuki and Diehl, 2017). To overcome the challenges in targeting a particular cellular site for NASH as a starting point and lack of knowledge of the safety information, we have taken the repurposing approach (Turner et al., 2016) and identified matrine as a potential new drug for the treatment of NASH based on its reported effects related on metabolic stress, inflammation, and fibrosis (Diehl and Day, 2017).

Matrine (Mtr, known as Kushen alkaloid) is originally isolated from the plant *Sophora flavescens* (Cheng et al., 2006) and it has been used as a hepatoprotective drug in China with minimal adverse effects (Liu et al., 2003). This small molecule drug (MW: 248) has been reported to inhibit inflammation (Zhang et al., 2011; Sun et al., 2016). Additionally, matrine has been shown to protect against liver damage in an ischemia-reperfusion rat model (Zhu et al., 2002). More recently, studies from our laboratory have demonstrated its therapeutic efficacy in eliminating hepatosteatosis resulting from either a high-fat diet or high-fructose diet in mice (Zeng et al., 2015; Mahzari et al., 2018). Importantly, these therapeutic effects appear to involve the heat shock protein (HSP) pathway but not those commonly recognized cellular targets such as PPARα, PPARγ, or AMPK (Zeng et al., 2015; Mahzari et al., 2018). However, those two mouse models lack the phenotypes of hepatic inflammation and fibrosis for us to evaluate its therapeutic effects for NASH (Friedman et al., 2018).

Therefore, the present study aimed to evaluate the effects of matrine on hepatic damage, inflammation and fibrosis in mice induced by a methionine choline-deficient (MCD) diet, a well-defined model of NASH (Diehl and Day, 2017). The mechanism involved were further investigated by examining associated changes in the HSP pathway and comparing with those produced by metformin, an anti-diabetic drug which has been suggested to have benefits for NASH (Clarke et al., 2015).

## Materials and Methods

### Animal Care and Experimental Design

Male C57BL/6J mice (10 weeks old) were purchased and acclimatized for at least 1 week. Mice were randomly assigned to four groups: feeding *ad libitum* with a standard chow diet (CH-Con; Gordon’s Specialty Stock Feeds, Yanderra, NSW, Australia); MCD alone (MCD-Con); MCD with matrine treatment as a food additive (MCD-Mtr; Mtr: 100 mg/kg/day); and MCD with metformin treatment as a food additive (MCD-Met; Met: 250 mg/kg/day) for 6 weeks. The doses of matrine and metformin were based used our previous publications (Zeng et al., 2015; Mahzari et al., 2018). Matrine (purity >99.5%) was a gift from Professor Li-Hong Hu from the Shanghai Institute of Materia Medica (China) and metformin was purchased from Sigma-Aldrich (Australia). The drug powders were stored at −20°C then weighted and carefully mixed with diet every day. All experiments were approved by the Animal Ethics Committee of RMIT University (#1415) in accordance with the guidelines of the National Health and Medical Research Council of Australia.

### Metabolic Assessments

Body weight and food intake were monitored daily throughout the experiment. Before the start of the study and at 5 weeks, following 5–7 h of food removal, tail vein blood was collected for glucose measurement with a glucometer (Accu-Check II; Roche, Castle Hill, Perth, Australia). Before the start of the study and at the end of the study, plasma was collected and stored at −80°C for subsequent biochemical testing. Mice were anesthetized with a ketamine/xylazine mixture (up to 100 mg/kg body weight ketamine and 20 mg/kg body weight xylazine) was administered via intraperitoneal injection. Mice fixation was then performed via transcardial perfusion with heparinized phosphate buffered saline (PBS; 10–20 mL/mouse) followed by 4% paraformaldehyde (PFA; 10–20 mL/mouse; #C007, ProSciTech). At the completion of the PFA perfusion, the right lobe of the liver was dissected and immersed in 4% PFA-filled glass scintillation vials for further analysis.

### Determination of Body Fat Composition and Liver Triglyceride Content

Total body fat content in mice was evaluated using the EchoMRI^TM^-100H body composition analyzer (EchoMRI). Mice were restrained live inside a tube during this harmless and non-invasive analysis. The principle of measuring the whole-body fat composition using the EchoMRI^TM^-100H was based on the magnetic resonance imaging (MRI) technique measuring live body composition such as fat tissue, lean tissue and free fluid. Hepatosteatosis was assessed by measuring TG contents in the liver using the method of Folch and a colorimetric assay kit (Triglyceride GPO-PAP; Roche, Castle Hill, NSW, Australia), as described previously (Ren et al., 2012).

### Assessment of the Effect on Liver Damage

To examine the effects of matrine on liver damage, the second component of NASH, plasma ALT, and AST were measured at baseline and at Week 6 using commercial kits (ALT/SGPT Liqui-UV; Australia) (Li et al., 2016). Food was removed from mice cages for 5–7 h, and blood was collected from the tail vein (50 μL blood + 50 μL saline; fast spin for 1 min), and then mixed with 200 μL reagent (R1:R2 = 5:1, as described in the manufacturer instructions). The absorbance was measured at 340 nm using a FlexStation (Molecular Devices, Australia). The total iron, and heme and non-heme iron, contents of liver samples were determined colorimetrically (Dang et al., 2011) because iron deposition is also regarded as a characteristic of NASH (George et al., 1998; Handa et al., 2016). As described previously (Riemer et al., 2004), the fixated liver tissue (50–60 mg) was homogenized with 50 mM NaOH (using suitable volume to provide uniform homogenisation). The sample was incubated overnight at 75–80°C, and then separated into two aliquots for determination of the total and non-heme iron contents. The total iron was determined by adding reagent A (a freshly mixed solution of equal volumes of 1.4 M HCl, 4.5% (w/v) KMnO_4_ and 40% TCA in H_2_O), and the non-heme iron was determined by adding reagent B (same as reagent A without KMnO_4_), to the sample (Owen et al., 2014). The iron content of the sample was calculated by comparing its absorbance to that of a range of standard concentrations of equal volume. Standards (five diluted standards) were prepared as a mixture of FeCl_3_ in 10 mM HCl and 50 mM NaOH. The standard value was measured using lysis reagent that either contained or lacked permanganate (Owen et al., 2014). The mixture was transferred into 96-well plates and its absorbance was measured at 550 nm.

### Real-Time Polymerase Chain Reaction of Liver RNA

Total RNA was isolated from mouse livers using TRIzol reagent (Invitrogen, #15596026) as previously described (Zeng et al., 2012). Real-time PCR was carried out using the IQ SYBR Green Supermix (Bio-Rad Laboratories Inc., United States) for genes of interest. Target gene expression was normalized to the housekeeping gene (18S). The primer sequence (5′ to 3′) of 18S is CGC CGCTAGAGGTGAAATTCT (sense) and CGAACCTCCGACTTTCGTTCT (antisense); TNFα: CACAAGATGCTGGGACAGTGA (sense) and TCCTTGATGGTGGTGCATGA (antisense); IL-1β: CAACCAACAAGTGATATTCTCCATG (sense) and GATCCACACTCTCCAGCTGCA (antisense); CD68: TGACCTGCTCTCTCTAAGGCTACA (sense) and TCACGGTTGCAAGAGAAACATG (antisense); Collagen 1: CTGCTGGTGAGAGAGGTGAAC (sense) and ACCAAGGTCTCCAGGAACAC (antisense).

### Western Blotting

Liver lysates were resolved by SDS-PAGE and immunoblotted with specific antibodies (Chan et al., 2013). Antibodies were diluted 1:500 (for HSF1, HSP72 and HSP90) or 1:1000 (for other antibodies) with a TBST buffer containing 1% BSA, 0.02% sodium azide, and 0.0025% phenol red (Sigma-Aldrich, Australia). Antibodies for HSP72 and HSP90 were purchased from Enzo Life Sciences (Farmingdale, United States); MCP-1, HSF1, TGFβ, Smad3, mammalian target of rapamycin (mTOR), phospho^Ser2448^ mTOR, α-Tubulin and GAPDH were purchased from Cell Signaling (Danvers, MA, United States). A nod-like receptor pyrin containing 3 (NLPR3) was purchased from AdipoGen (San Diego, United States). Goat Anti Mouse, Goat Anti Rabbit and Goat Anti-Rat from Santa Cruz (United States). Proteins were quantified using a ChemiDoc, and densitometry analysis was performed using Image Lab software (Bio-Rad Laboratories, Australia).

### Histological Evaluation of Liver Sections

The liver samples were perfused using PFA and sliced into 5 μm sections. Free-floating sections were stained with picrosirius red for liver fibrosis, and then quantified in five non-overlapping fields of view per animal by using an Olympus BX41 microscope with a 20× objective lens and an Olympus DP72 digital camera (Olympus, Australia) (Witek et al., 2009; Li et al., 2016). The mean value was calculated for each experimental group using the threshold function in the ImageJ software package (NIH, Bethesda, MD, United States). Data are represented as percentage (%) of positive area per field. To obtain statistical significance, at least five random-field images were taken per slide, and at least seven mice per group were scored (*n* = 7).

### Statistical Analysis

All results are presented as means ± SEM. One-way analysis of variance was used to assess the statistical significance across all groups. When significant differences were found, the Tukey-Kramer multiple comparisons *post hoc* test was used to establish differences between groups. Differences at *p* < 0.05 were considered statistically significant and *p* < 0.01 were considered highly significant.

## Results

### Effects on Adiposity, Hepatosteatosis, and Plasma Glucose

Methionine choline-deficient diet feeding is a common dietary model of NASH despite the absence of several metabolic phenotype on body weight gain and calorie intake (Rinella and Green, 2004; Hebbard and George, 2011). As expected, MCD diet-fed mice showed a reduced body weight, body weight gain, and fasting blood glucose level despite a significant increase in calorie intake. As shown in Table 1, the excess TG accumulation, indicative of hepatosteatosis, was increased dramatically in the liver of MCD-Con mice compared with CH-Con (by twofold, *p* < 0.01). The difference in weight gain between chow, MCD alone and MCD with treatments was maintained throughout the 6-week treatment period.

**Table 1:** Effects on body weight, total food intake and liver triglyceride.

	CH-Con	MCD-Con	MCD-Mtr	MCD-Met
Body weight (g)	24.3 ± 0.2	23.5 ± 0.2^∗∗^	22.6 ± 0.1^††^	22.7 ± 0.1^††^
Body weight gain (g)	2.0 ± 0.2	0.4 ± 0.2^∗∗^	0.3 ± 0.2	0.7 ± 0.1
Caloric intake (kcal/kg.day)	268 ± 8	314 ± 9	320 ± 10	335 ± 18
Fasting blood glucose (mM)	8.0 ± 0.5	7.4 ± 0.4	6.3 ± 0.4	6.4 ± 0.5
Liver triglyceride (μmol/g)	13.8 ± 4.2	31.6 ± 3.3^∗∗^	28.2 ± 2.5	31.2 ± 3.1

*Mice were fed a chow (CH-Con), or MCD alone (MCD-Con), MCD-treated with matrine (MCD-Mtr; 100 mg/kg/day) or MCD-treated with metformin (MCD-Met; 250 mg/kg/day). Body weight and food intake were measured twice a week. Blood glucose was performed at week 5. Plasma was collected before tissue collection at week 6 for the subsequent analysis for ALT and AST. ^∗∗^p < 0.01 vs. CH-Con; ^††^p < 0.01 vs. MCD-Con (n = 8 mice/group).*

Matrine had no effect on body weight gain and calorie intake in MCD diet-fed mice. Consistent with previous studies (Rinella and Green, 2004; Machado et al., 2015), MCD diet-fed mice showed increased adiposity and hepatosteatosis but no glucose intolerance or mild hypoglycemia. There was no change in fasting blood glucose level in matrine-treated MCD diet-fed mice compared to MCD diet-fed group. Associated with these effects, matrine also was had no effect in reducing hepatic TG in MCD diet-fed mice (Table 1). Similarly, metformin did not show any effect on any of these parameters in MCD diet-fed mice.

### Effects on Body Composition Using MRI

**FIGURE 1:**
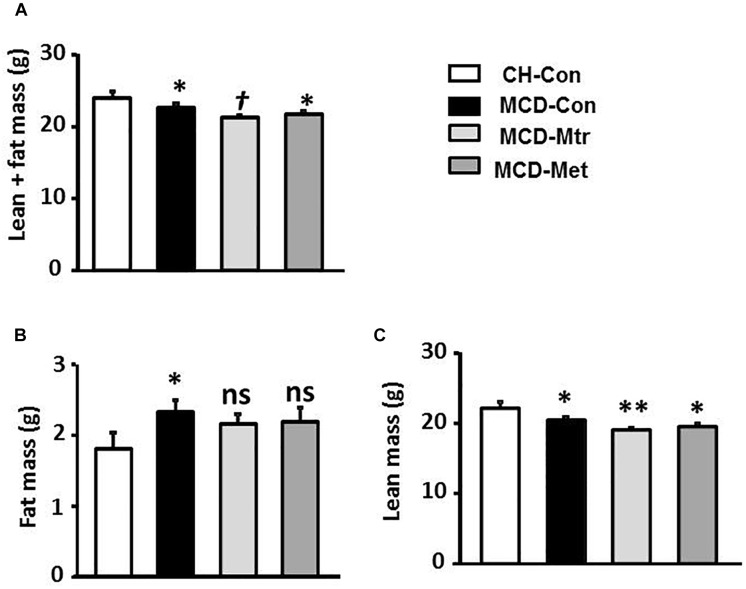
Effects of Mtr on body composition in MCD mice. The body composition of CH-Con, MCD-Con, MCD-Mtr, and MCD-Met was determined by EchoMRI analyzer at week 4 **(A)** Lean+fat mass; **(B)** Fat mass; **(C)** Lean mass. ^∗^*p* < 0.05, ^∗∗^*p* < 0.01 vs. CH-Con; *^†^p* < 0.05 vs. MCD-Con; ns, not significant vs. CH-Con or MCD-Con (*n* = 8 mice/group).

Methionine choline-deficient diet feeding resulted in a lack of metabolic phenotype possibly due to significantly decreased body weight (Figure 1A). Total body fat content using the EchoMRI analyzer was measured in all mice. As illustrated in Figure 1B, in spite of the reduction in body weight by MCD diet feeding, there was a significant increase in fat mass in MCD diet-fed mice compared with the chow-fed group. MRI analysis also revealed that MCD diet-fed mice and mice treated with either matrine or metformin had a significant decrease in lean mass compared with the chow group (Figure 1C). However, matrine and metformin had no effect on the fat mass.

### Effects on Hepatic Inflammation

An important pathological characteristic of NASH is hepatic inflammation, and MCD diet has previously been shown to cause marked hepatic inflammation (Chalasani et al., 2012; Santhekadur et al., 2018). Key inflammatory proteins, including TNFα, IL-1β, MCP-1, cluster of differentiation 68 (CD68), and NLRP3 inflammasome, are particularly associated with liver inflammation and the progression of NASH (Farrell et al., 2012; Arab et al., 2018). To examine the anti-inflammatory effects of matrine in MCD diet-fed mice, the protein expression levels of these inflammatory markers was measured. Consistent with other studies (Rinella et al., 2008; Sutti et al., 2014; Mridha et al., 2017), MCD feeding resulted in a marked inflammatory response in the liver as evidenced by increased expression levels of TNFα and CD68 ( ± 50 and 80%, respectively; both *p* < 0.05), an indicator of KCs activation (Tacke, 2017). Further, livers of MCD-Con mice with NASH exhibited a substantial increase in MCP-1 and NLRP3 expression (both 38% vs. CH-Con).

**FIGURE 2:**
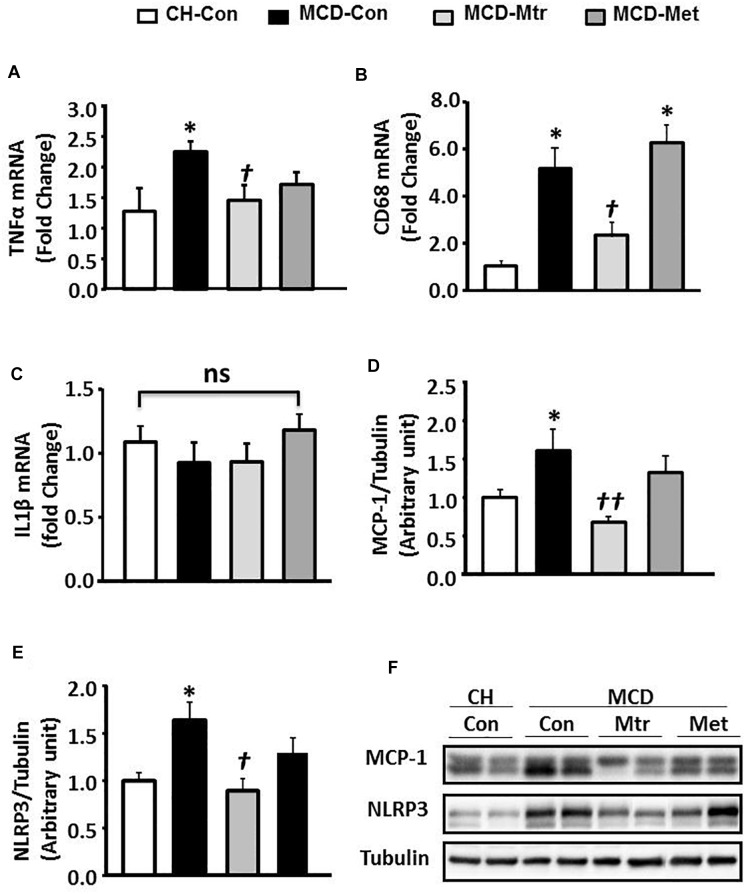
Effects of Mtr on inflammation in MCD-fed mice. **(A)** TNFα mRNA, **(B)** CD68 mRNA, **(C)** IL1β mRNA, **(D)** MCP-1, **(E)** NLRP3, and **(F)** representative western blot images. ^∗^*p* < 0.05 vs. CH-Con; *^†^p* < 0.01, *^††^p* < 0.01 vs. MCD-Con; ns, not significant (*n* = 7–8 mice/group).

Matrine treatment normalized the expression levels of TNFα, CD68 (both *p* < 0.05 vs. MCD-Con) and MCP-1 (*P* < 0.01 vs. MCD-Con), as shown in Figure 2A,B,D. However, no significant differences were detected in the expression of IL-1β among the experimental groups (Figure 2C). It has been suggested that NLRP3 blockade reverses advanced stage liver inflammation and fibrosis in MCD diet-induced NASH (Mridha et al., 2017). Consistent with the previous study, there was ± 65% reduction in NLRP3 inflammasome expression (*P* < 0.05 vs. CH-Con) (Figure 2E). In contrast to matrine, metformin had no effect on the expression levels of inflammatory proteins including TNFα, CD68, MCP-1, and NLRP3 (Figure 2A–F).

### Effects on the Hepatic Fibrogenesis

**FIGURE 3:**
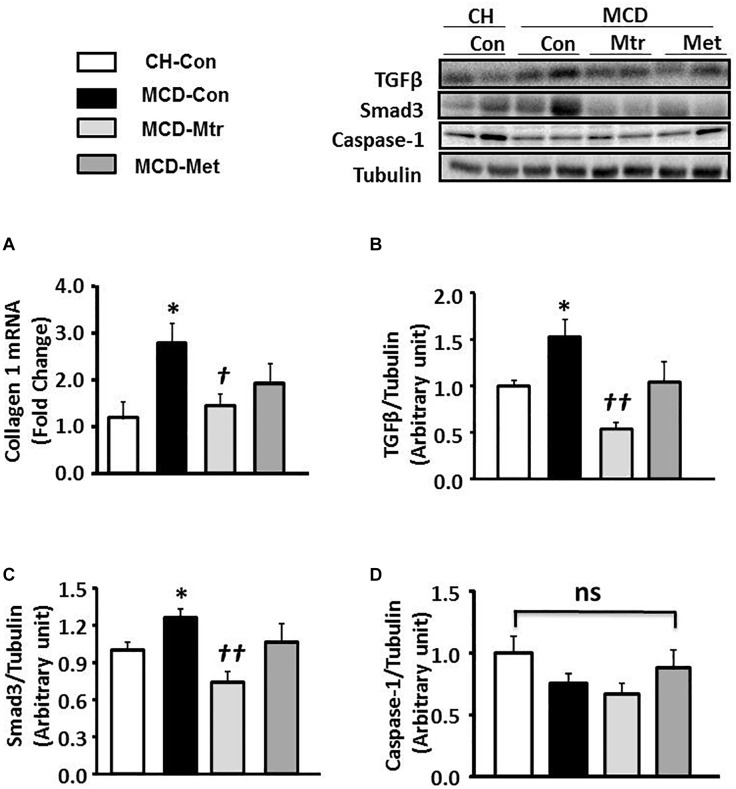
Effects of Mtr on fibrosis in MCD diet-fed mice. **(A)** A hepatic fibrosis gene of collagen 1 was determined by quantitative RT-PCR analysis. Liver lysates from mice were immunoblotted for **(B)** TGFβ, **(C)** Smad3, and **(D)** caspase-1 and quantified for statistical analysis. ^∗^*p* < 0.05 vs. CH-Con; *^†^p* < 0.05, *^††^p* < 0.01 vs. MCD-Con; ns, not significant (*n* = 7–8 mice/group).

Liver fibrosis is another hallmark of advanced NASH (Suzuki and Diehl, 2017). As shown in Figure 3A–C, MCD diet-fed mice exhibited marked increases in the expression of key proteins of the pro-fibrotic pathway in the liver, namely collagen 1, TGFβ and Smad3 (all *p* < 0.05 vs. CH-Con), and these results were consistent with others (Yamaguchi et al., 2007; Mridha et al., 2017). Treatment of MCD diet-fed mice with matrine inhibited hepatic expression of these proteins ( ± 55, 65, and 45%, respectively) toward the levels seen in CH-Con mice. In contrast, metformin treatment had no effect on MCD diet-induced liver fibrosis. There were no significant changes in caspase-1 protein expression between groups (Figure 3D). These results revealed that treatment with matrine attenuated MCD diet-induced fibrosis in the liver.

**FIGURE 4:**
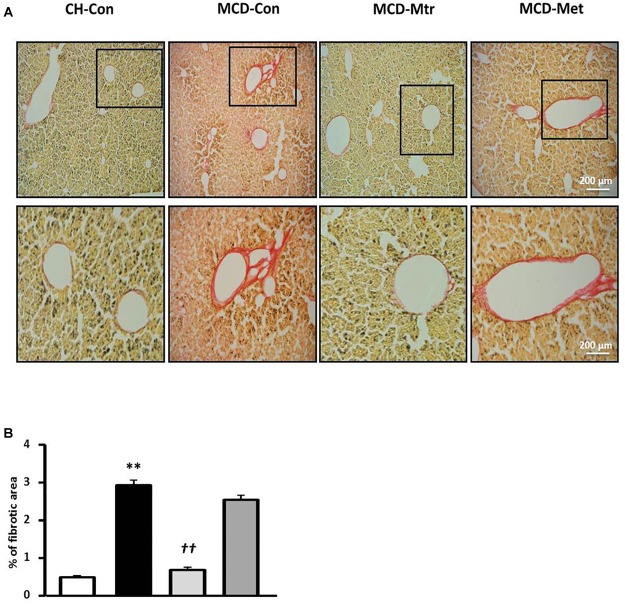
Effects of Mtr on fibrosis in MCD diet-fed mice. **(A)** Representative images showing collagen staining with picrosirius red in liver sections from CH-Con, MCD alone, and MCD treated groups [scale bar = 200 μm (top set) and 50 μm (below set), 10× magnification]. **(B)** Mtr treated mice had significantly reduced fibrosis area compare to MCD-Con group. ^∗∗^*p* < 0.01 vs. CH-Con; *^††^p* < 0.01 vs. MCD-Con (*n* = 7–8 mice/group).

To further evaluate the effect of matrine on hepatic fibrogenesis, liver sections were stained with picrosirius red to quantify the extent of liver fibrosis. As shown in Figure 4, there was ∼83% increase of liver fibrosis (*p* < 0.01 vs. CH-Con fed mice) in MCD-fed mice and this increase was reversed following treatment with matrine (∼66% reduction, *p* < 0.01 vs. MCD-Con mice). In comparison, no significant reduction of liver fibrosis was observed following metformin treatment.

### Effects on Plasma Level of Liver Enzymes and Iron Deposition

**FIGURE 5:**
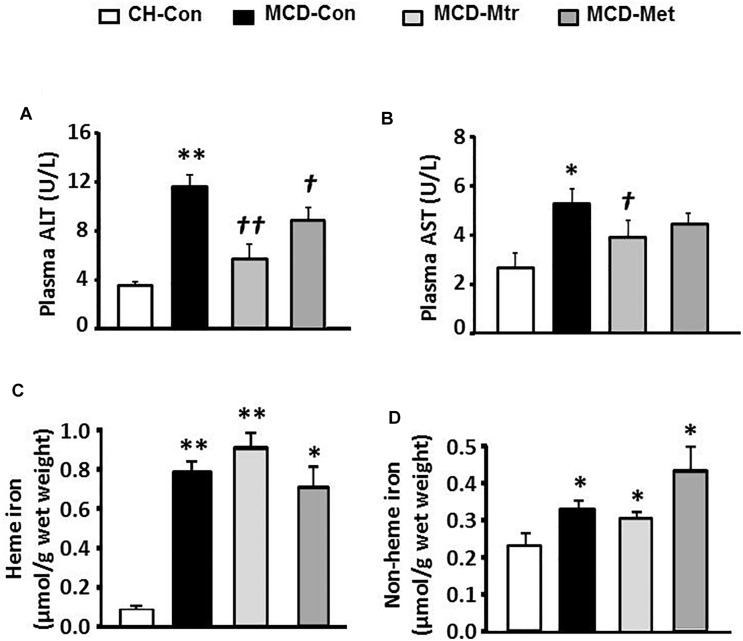
Effects of Mtr on liver damage and iron level. After 6 weeks of feeding and drug treatment, blood samples were collected for the measurement of **(A)** ALT and **(B)** AST levels. Liver tissue was collected for iron measurement **(C)** heme iron and **(D)** non-heme iron. ^∗^*p* < 0.05, ^∗∗^*p* < 0.05 vs. CH-Con; *^†^p* < 0.05, *^††^p* < 0.01 vs. MCD-Con (*n* = 7–8 mice/group).

Liver damage has been suggested to be an important factor that distinguishes NASH from hepatosteatosis (Reid et al., 2016). We next examined whether matrine treatment may alleviate liver damage in MCD diet-fed mice. As shown in Figure 5A, MCD diet-fed mice exhibited marked increases in ALT (by twofold) and AST (by onefold). Treatment with matrine or metformin markedly decreased ALT levels ( ± 80 and 25%, respectively). In addition, matrine treatment but not metformin, further decreased AST levels (*p* < 0.05) in MCD diet-fed mice, indicating matrine may reduce liver damage, a hallmark in the progression of NASH.

We further examined the effects of matrine on iron levels in the liver of MCD-fed mice. Neither matrine nor metformin treatment altered the accumulation of heme and non-heme iron induced by MCD diet (Figure 5B). Neither matrine nor metformin treatment altered the accumulation of heme and non-heme iron induced by MCD diet (Figure 5C,D). This was likely due to an excessive overload of iron in the liver among mice fed MCD diet.

### Effects on Hepatic mTOR and HSPs Expression

**FIGURE 6:**
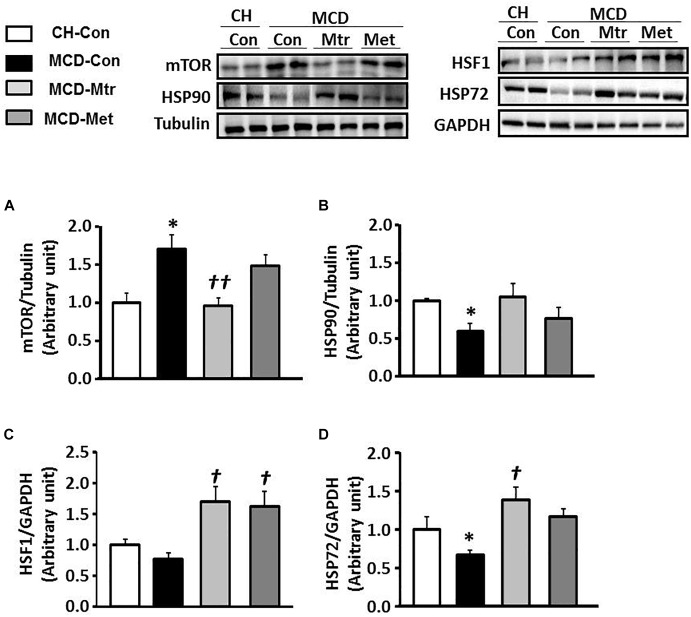
Effects of Mtr on HSF1, HSP90, HSP72, and mTOR in MCD diet-fed mice. Liver lysates from mice were immunoblotted for **(A)** mTOR **(B)** HSP90, **(C)** HSF1, and **(D)** HSP72 and quantified for statistical analysis. ^∗^*p* < 0.05 vs. CH-Con; *^†^p* < 0.01, *^††^p* < 0.01 vs. MCD-Con (*n* = 7–8 mice/group).

Dysregulation of mTOR signaling has been implicated in fatty liver diseases (Han and Wang, 2018). Inhibition of mTOR has previously been shown to be efficacious in improvement of NASH-induced by MCD diet (Sapp et al., 2014; Chen et al., 2016; Gong et al., 2016). To elucidate the mechanism underlying mTOR inhibition-induced by matrine, protein expression using western blotting was performed to evaluate whether mTOR contributes to hepatocyte damage, inflammation, and fibrosis in NASH. Interestingly, treatment with matrine normalized the protein level of mTOR (*p* < 0.01 vs. MCD-Con) toward the levels seen in CH-Con mice (Figure 6A). These results revealed that treatment with matrine inhibited MCD diet-induced hepatic mTOR expression. To determine whether matrine treatment is associated with the upregulation of HSP-induced improvement in NASH, HSP90, HSF1, and HSP72 expression was measured in the liver. As shown in Figure 6B–D, HSP90 and HSP72 expression levels were blunted (50% reduction vs. CH-Con, *p* < 0.05) by MCD diet feeding, but there was no significant effect on the protein levels of HSF1. Treatment with matrine upregulated hepatic HSP72 and HSF1 expression levels (*p* < 0.01 vs. MCD-Con) in MCD diet-fed mice, but had no significant effect on HSP90 expression. Therefore, these results indicated that the beneficial effect of matrine on the development of MCD diet-induced NASH might be associated with its ability to limit mTOR and upregulate HSPs. In comparison, metformin treatment significantly rescued HSF1 but had no significant effect on the expression of HSP72 and HSP90.

## Discussion

Our previous studies, which focused on the metabolic effect of matrine, have demonstrate that matrine treatment was effective in reducing hepatosteatosis, adiposity and glucose intolerance in HFru diet-induced mouse models (Mahzari et al., 2018). Current study further investigates the effect of matrine on NASH, a hepatic manifestation of the metabolic syndrome (Diehl and Day, 2017). This study demonstrated that matrine markedly ameliorated hepatic damage, inflammation and fibrosis with inhibition of mTOR and upregulation of HSP72, in a mouse model of NASH induced by MCD diet feeding. In comparison, metformin had no effect on inflammation, collagen deposition, HSP72 or mTOR.

While high-fat or high-fructose feeding results in several prominent features of metabolic syndrome (including obesity, hepatosteatosis, glucose intolerance etc.), they do not induce apparent and severe hepatic damage or fibrosis in the liver (Santhekadur et al., 2018). In contrast, MCD diet feeding is a well-recognized model of NASH that rapidly induces hepatic steatosis, damage, inflammation, and fibrosis in mice, despite of the absence of insulin resistance and hyperglycemia (Rinella and Green, 2004; Hebbard and George, 2011; Santhekadur et al., 2018). Therefore, we evaluated the protective effects of matrine on NASH in mice fed a MCD diet.

Consistent with previous studies, mice fed a MCD diet developed hepatic inflammation and fibrosis that reflected the natural course of NASH in human (Rinella and Green, 2004; Rinella et al., 2008; Machado et al., 2015). Matrine prevented MCD diet-induced inflammation and fibrosis in the liver after 6 weeks of treatment, as demonstrated by a matrine-induced suppression of the increases in TNFα, CD68, MCP-1 and NLRP3, and hepatic fibrosis proteins (TGFβ, Smad3, and collagen 1) induced by MCD diet.

Several studies indicated that activation of inflammatory cytokines has a vital role in the progression of NASH (Diehl and Day, 2017; Schuppan et al., 2018). The increase of pro-inflammatory cytokines production via the activation of Kupffer cells, in particular TNFα, was suggested to be the key mediator of NASH progression (Tosello-Trampont et al., 2012). Alternatively, inhibition of TNFα activity using anti-inflammatory drugs improved liver damage, inflammation, and NASH (Li et al., 2003; Koppe et al., 2004). Furthermore, in line with increased TNFα production, the increased presence of CD68 and MCP-1 are associated with the severity of NASH (Li et al., 2016; Diehl and Day, 2017). The results of the current study proved that matrine significantly reduced these inflammatory targets-induced by MCD diet feeding. Another central participator in the development of NASH is the activation of the NLRP3 inflammasome (Henao-Mejia et al., 2012; Wree et al., 2014). It has been recently reported that blockage NLRP3 activation reduced liver inflammation and fibrosis in MCD diet-fed mice (Mridha et al., 2017). It generally believes that the anti-inflammatory activities of matrine are largely due to its ability to scavenge airway inflammation and hepatic inflammation (Zhu et al., 2002). The result of matrine-induced attenuation of hepatic inflammation, through inhibiting the activity of TNFα and suppressing several inflammatory proteins and chemokines (including CD68, MCP-1, and NLRP3) in the MCD diet-fed mice, supported the notion that matrine is likely to reduce NASH via inhibition of the inflammatory pathway.

Consistent with elevated levels of pro-inflammation cytokines and exacerbated fibrosis, MCD-diet feeding resulted in hepatic fibrosis, is also known as another hallmark of NASH (Rinella and Green, 2004; Diehl and Day, 2017), and it is considered as a result of repetitive inflammation events (Schuppan et al., 2018). In NASH, fibrosis closely correlates with the degree of inflammation and severity of the disease (Tsuchida and Friedman, 2017; Schuppan et al., 2018). While matrine prevents liver fibrosis (TGFβ and collagen production) induced by carbon tetrachloride (CCl_4_) in rats (Zhang et al., 2001), whether matrine inhibit fibrosis in a NASH model without the involvement of chemical toxicity remains unclear. To investigate whether the inhibition of hepatic inflammation by matrine involves the inhibition of fibrosis signaling and collagen production (Itagaki et al., 2013; Wree et al., 2014); we examined the effect of matrine on fibrosis in a mouse NASH model induced by MCD diet. Consistent with the reduction in inflammation, our data indicated that matrine inhibited the activation of fibrosis by suppression of TGFβ, Smad3 and collagen 1 synthesis in the liver of MCD diet-fed mice. The effect of matrine in alleviating fibrosis was further support by the histological data revealing a significant reduction in the number of collagen proportionate area in MCD diet-fed mice after matrine treatment.

Consistent with elevated levels of pro-inflammation cytokines and exacerbated fibrosis, MCD-diet feeding resulted in severe liver damage as indicated by the elevated plasma level of liver enzymes, particularly ALT, which has been considered as a requisite in the diagnosis of NASH (Torres and Harrison, 2013; Diehl and Day, 2017). Co-administration of matrine prevented the elevated plasma levels of ALT and AST, indicating a less extent of liver damage, which is in line with the effects of matrine on hepatic inflammation and fibrosis.

After demonstrating the effect of matrine in improving inflammation, fibrosis and liver damage in MCD diet-induced NASH, we next investigated the possible molecular mechanism involved. Our previous work suggested that upregulation of HSP72 may contribute to hepatosteatosis induced by HFD or HFru feeding in mice (Zeng et al., 2015). Another study from our lab showed that matrine could reduce glucose intolerance in mice caused by an increase in *de novo* lipogenesis (DNL) in HFru diet-fed mice (Mahzari et al., 2018). The present study found that mice fed with matrine-MCD diet showed significantly higher concentrations of HSP72 and HSP90 protein in the liver. Heat shock protein 72 (HSP72) is a major inducible HSP, which exerts cytoprotective effects by assisting in protein folding, protein degradation, signal transduction and translocation of client proteins across membranes (Hartl et al., 2011). It is been suggested that HSP72 expression is progressively suppressed in the liver and muscle of obese and NAFLD patients (Di Naso et al., 2015), and in skeletal muscle of T2D patients (Kurucz et al., 2002). An elevation in HSP72 is associated with the reduction in JNK phosphorylation and attenuation of insulin resistance (Chung et al., 2008). These results suggest the anti-inflammatory effects of matrine, at least in part, involving the activation of HSP72 in the liver.

It is worthwhile noting that matrine also inhibited MCD-induced expression of mTOR. This is interesting because overexpression of mTOR contributes to NAFLD progression (Wang et al., 2014). It has been suggested to play an important role in the development of NASH by activating inflammation and fibrosis in mice fed with choline-deficient diet-induced steatohepatitis (Gong et al., 2016). In comparison, metformin showed no effect on MCD-induced increase in mTOR or NASH phenotype. These data suggest that the suppressed mTOR by matrine may also contribute to its therapeutic effects for NASH.

Previous studies in cultured cell suggest that the PI3K/Akt/mTOR signaling can mediate the upregulation of HSP72 (Ma et al., 2015) and potentiate the upregulation of HSP72 by HSP90 inhibitors (Acquaviva et al., 2014). However, it has also been reported that overexpression of HSP72 may activate the mTOR pathway under certain conditions (Dokladny et al., 2013). Our own work suggests that matrine has a high binding affinity at HSP90 and possibly induce the upregulation of HSP72 by inhibiting HSP90 (Zeng et al., 2015) as widely recognized (Acquaviva et al., 2014). Further studies are required to determine the causal relationship between HSP72 and mTOR (including their activity and downstream changes in autophagy) and its role in the molecular of action in protective effects of matrine against the development of MCD-induced NASH.

**FIGURE 7:**
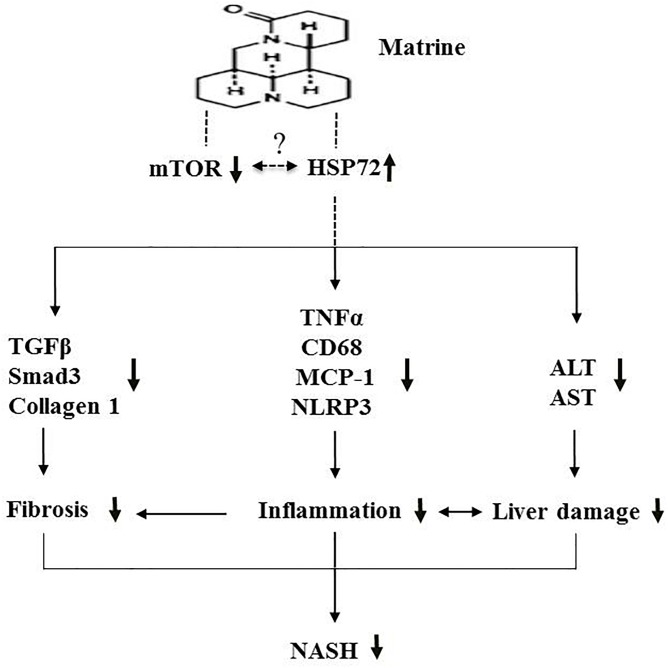
A schematic diagram illustrating the proposed mechanism underlying the effects of Mtr against MCD diet-induced NASH.

In conclusion, the present study indicates that the hepatoprotective drug matrine may offer protective effects against NASH via suppression of hepatic inflammation, fibrosis and damage, possibly via the upregulation of HSP72 and the downregulation of mTOR (Figure 7). Further investigation of the therapeutic effects of matrine for more advanced NASH is well warranted.

## Ethics Statement

The animal ethics has been approved by the RMIT University Animal Ethics Committee (AEC) for the period from August 21, 2014 until August 20, 2017. The animal ethics number is AEC 1415 and the title of the project is therapeutic effects of matrine on non-alcoholic steatohepatitis (NASH) in mice.

## Author Contributions

J-MY and AM conceived/designed the study and analyzed the data. AM, SL, XZ, SF, MA, and WA performed the experiments. SR and DL provided intellectual input and technical supports. AM drafted the manuscript. J-MY and XZ revised the manuscript.

## Conflict of Interest Statement

The authors declare that the research was conducted in the absence of any commercial or financial relationships that could be construed as a potential conflict of interest.

## Abbreviations

ALT: alanine transaminase
AST: aspartate aminotransferase
HSF1: heat shock factor 1
HSP72: heat shock protein 72
HSP90: heat shock protein 90
Mtr: Matrine
MCD: methionine choline-deficient
Met: metformin
mTOR: mammalian target of rapamycin
NAFLD: non-alcoholic fatty liver disease
NASH: non-alcoholic steatohepatitis
TGF-β: transforming growth factor β.
